# Low neutrophil-to-lymphocyte and platelet-to-lymphocyte ratios predict favorable outcomes after endovascular treatment in acute basilar artery occlusion: subgroup analysis of the BASILAR registry

**DOI:** 10.1186/s12883-023-03161-2

**Published:** 2023-03-20

**Authors:** Jia sheng Liao, Changwei Guo, Bo Zhang, Jie Yang, Wenjie Zi, Jing lun Li

**Affiliations:** 1 Department of Neurology, The Affiliated Hospital of SouthWest Medical University, No. 25, Taiping Street, Jiangyang District, Luzhou City, 646000 China; 2 Department of Neurology, Xinqiao Hospital and The Second Affiliated Hospital, Army Medical University (Third Military Medical University), Chongqing, 400037 China; 3 Department of Cerebrovascular Diseases, Suining First People’s Hospital, Suining, 629000 China

**Keywords:** Neutrophil-to-lymphocyte ratio, Platelet-to-lymphocyte ratio, Basilar artery occlusion, Endovascular treatment

## Abstract

**Background:**

Recently, the BAOCHE trial and ATTENTION trial registry have demonstrated the efficacy of endovascular treatment (EVT) in patients with acute basilar artery occlusion (BAO), however, the proportion of patients with favorable post-EVT outcomes remains low. The present study aimed to investigate the individual and joint prognostic values of the neutrophil-to-lymphocyte ratio (NLR) and platelet-to-lymphocyte ratio (PLR) in patients with acute BAO who have undergone EVT.

**Methods:**

We enrolled patients who underwent EVT from the BASILAR registry. Patients were divided into the following groups based on their modified Rankin Scale (mRS) scores at 90 days: favorable-outcome (mRS score: 0–3) and poor-outcome (mRS score: 4–6) groups. Multivariable logistic regression was performed to analyze the association of NLR and PLR with favorable post-EVT outcomes.

**Results:**

In total, 585 patients with EVT were recruited. Of these, 189 and 396 patients were in the favorable-outcome and poor-outcome groups, respectively. According to the multivariable logistic regression analyses, both NLR (adjusted odds ratio [aOR], 0.950; 95% confidence interval [CI], 0.920–0.981; *P* = 0.002) and PLR (aOR, 0.997; 95% CI, 0.995–0.999; *P* = 0.002) were related to favorable post-EVT outcomes in patients with acute BAO. The optimal cutoff values for the NLR and PLR were 7.75 and 191, respectively. Furthermore, stratified analysis using the multivariable logistic regression model revealed that both NLR and PLR (NLR values ≥ 7.75 and PLR values ≥ 191) were associated with a low rate of favorable outcomes (aOR, 0.292; 95% CI, 0.173–0.494; *P* < 0.001).

**Conclusions:**

Low NLR and PLR were both associated with favorable post-EVT outcomes in patients with acute BAO. Furthermore, the combined value of both inflammatory markers is potentially reliable in predicting clinical post-EVT outcomes.

**Supplementary Information:**

The online version contains supplementary material available at 10.1186/s12883-023-03161-2.

## Introduction

Acute basilar artery occlusion (BAO) is a catastrophic and infrequent disease associated with severe disability and high mortality; moreover, it is associated with the gravest form of posterior circulation strokes [1, 2]. The Basilar Artery Occlusion Chinese Endovascular (BAOCHE) [3] trial, the Endovascular Treatment for Acute Basilar Artery Occlusion (ATTENTION) [4] trial registry, and several observational studies have reported the efficacy of endovascular treatment (EVT) in patients with acute BAO [5, 6]. Nevertheless, the proportion of patients with favorable post-EVT outcomes remains low, predominantly ranging from 32 to 42% [6–8]. On this premise, identifying key predictive factors related to favorable post-EVT outcomes in patients with acute BAO is indispensable.

Post-ischemic inflammation, which causes worsening neurological dysfunction and secondary brain injury, has been found to be an important pathological process in various stages of cerebral ischemic injury, coinciding with the activation of intravascular leukocytes; stagnant blood flow; and the release of proinflammatory mediators from the platelet granules, ischemic endothelium, and brain parenchyma [9–11]. White blood cell count is an easily obtainable and the most widely used inflammatory biomarker in clinical practice. Previous studies have indicated that low lymphocyte and high neutrophil counts are associated with poor outcomes of acute cerebral infarction, and platelets have been found to play a pivotal role in thrombogenesis and inflammation [12, 13].

The neutrophil-to-lymphocyte ratio (NLR) and platelet-to-lymphocyte ratio (PLR) have recently been suggested as potential novel biomarkers in systematic inflammation and been demonstrated to possess predictive and diagnostic capabilities in several diseases [14, 15]. A previous retrospective study reported that the NLR value was associated with stroke severity, poor outcome, and stroke recurrence in patients with acute ischemic stroke [16]. Altintas et al. [17] found a high PLR value to potentially increase the proportion of poor outcomes and futile recanalization in patients with acute ischemic stroke (AIS) who have undergone EVT. Moreover, prior studies have shown that these two novel composite inflammatory ratios exhibit a superior predictive capacity to traditional inflammatory factors [18]. However, the relationship between these composite inflammatory ratios and favorable post-EVT outcomes in patients with acute BAO remains uncertain.

In this study, we analyzed data from the Endovascular Treatment for Acute Basilar Artery Occlusion Study (BASILAR) to explore the individual and joint prognostic values of the NLR and PLR in patients with acute BAO who have undergone EVT.

## Methods

### Study design and patient selection

In our study, patients with acute BAO who received EVT in our study were recruited from the BASILAR registry, a multicenter, observational, prospective registry that includes 47 comprehensive stroke centers across 15 provinces in China from January 2014 to May 2019. This analysis of human subjects was approved by the Ethics Committee of Xinqiao Hospital (Second Affiliated Hospital), Army Medical University Board. The detailed protocol of this study has previously been published [7]. All participants or their authorized representatives provided written informed consent in line with the principles of the Declaration of Helsinki. The inclusion and exclusion criteria were based on the BASILAR registry.

### Data collection

We collected data on demographics, vascular risk factors, prior thrombolysis history, time metrics, laboratory examinations, stroke etiology from the Trial of Org 10172 in Acute Stroke Treatment [19], and initial-stroke neurological-deficit severity assessed using the National Institutes of Health Stroke Scale (NIHSS). Angiographic images, including occlusion sites, the posterior circulation collateral score (PC-CS), and the posterior-circulation Alberta Stroke Program Early Computed Tomography Score (pc-ASPECTS), were independently assessed by two neuroradiologists who were blinded to the treatment assignments, clinical data, and outcomes. In case of discrepancies, another experienced neuroradiologist made the final decision.

All blood cell samples, including neutrophil, lymphocyte, and platelet counts, were obtained from each patient at the time of hospitalization before undergoing EVT for the examination of laboratory parameters. Thereafter, the cell counts were analyzed using an auto-analyzer (XE-2100, Sysmex, Kobe, Japan), and composite inflammatory ratios (including the NLR and PLR) were subsequently calculated. The NLR and PLR were computed as the ratio of the neutrophil to lymphocyte count and that of the platelet to lymphocyte count, respectively.

Finally, to achieve the aim of the present study, we assessed 90-day functional outcomes using the modified Rankin Scale (mRS) score as evaluated by trained and experienced local neurologists. The mRS is a 7-point scale ranging from 0 (no symptoms) to 6 (mortality) [20]. Based on their the 90-day mRS scores, patients were divided into favorable-outcome (mRS score: 0–3) and poor-outcome (mRS score: 4–6) groups.

### Endovascular treatment

All the patients in our study received EVT, which included mechanical thrombectomies with thromboaspiration, stent retrievers, balloon angioplasties, stenting, intra-arterial thrombolysis, or combinations of these approaches [7, 21]. The EVT method was selected by the treating neuro-interventionalists.

### Statistical analysis

Regarding baseline characteristics, categorical variables are presented as counts and proportions. Continuous variables were tested for distribution normality using the Kolmogorov–Smirnov test; means and standard deviations (SDs) have been used to describe normally distributed continuous variables, while medians and interquartile ranges (IQRs) have been employed to describe nonnormally distributed continuous variables. Univariate analysis was performed using the two independent samples t-test or Mann–Whitney U-test, chi-square test, or Fisher’s exact test, as appropriate.

We evaluated the relative effect of NLR and PLR values on favorable outcomes and mortality using multivariable logistic regression with adjustments for the following confounders: age, diabetes mellitus, puncture-to-recanalization time, baseline NIHSS score, baseline pc-ASPECTS score, occlusion sites, and stroke etiology. Due to the sample size and collinear factors, we chose adjusted confounders according to the previous literature reports and factors in the Table 1*P* < 0.05. Unadjusted and adjusted odds ratios (ORs) and their 95% confidence intervals (CIs) were calculated for four patient groups based on the IQRs of their NLR and PLR values (with the lowest group as a reference). A receiver operating characteristic (ROC) curve and Youden’s J statistic were used to determine the cutoff value for NLR and PLR. 7.75 and 191 were the cutoff value of NLR and PLR, respectively. According to the optimal cutoff values of the NLR and PLR, all the recruited patients in our study were divided into four groups (NLR value ≥ 7.75 and PLR value ≥ 191, NLR value ≥ 7.75 and PLR value < 191, NLR value < 7.75 and PLR value ≥ 191, and NLR value < 7.75 and PLR value < 191). Kaplan–Meier curve analysis was used to determine the cumulative probabilities of survival in the four groups during the 1-year follow-up.

**Table 1:** Baseline Characteristics in patients with favorable and poor outcomes

	Overall *n* = 585	Favorable outcome *n* = 189	Poor outcome *n* = 396	*P* value*	*P* value
Age, y, median (IQR)	64 (56–73)	63 (55–71)	65 (57–73)	0.09	0.06
Male, n/total n (%)	442 (75.6)	138 (73.0)	304 (76.8)	0.21	0.32
Medical history, n/total n (%)					
Hypertension	402 (68.7)	128 (67.7)	274 (69.2)	0.12	0.72
Hyperlipidemia	195 (33.3)	68 (36.0)	127 (32.1)	0.37	0.35
Diabetes mellitus	134 (22.9)	29 (15.3)	105 (26.5)	0.05	0.003
Coronary heart disease	92 (15.7)	21 (11.1)	71 (17.9)	0.34	0.03
Atrial fibrillation	121 (20.7)	45 (23.8)	76 (119.2)	0.91	0.20
Stroke	140 (23.9)	37 (19.6)	103 (26.0)	0.74	0.09
Smoking	212 (36.2)	72 (38.1)	140 (35.4)	0.66	0.52
Blood pressure on admission, mmHg, median (IQR)					
Systolic	149 (133–166)	146 (130–161)	150 (134–168)	0.09	0.048
Diastolic	84 (76–97)	84 (76–95)	85 (77–98)	0.03	0.29
Neutrophils, median (IQR)	9.14 (6.60–11.94)	7.80 (5.96–10.22)	9.82 (7.30–12.50)	< 0.001	< 0.001
Lymphocytes, median (IQR)	1.13 (0.80–1.60)	1.23 (0.90–1.65)	1.08 (0.78–1.55)	0.05	0.01
Platelets, median (IQR)	211 (173–252)	1.08 (0.78–1.55)	215 (174–254)	0.07	0.18
NLR, median (IQR)	7.92 (4.96–12.59)	6.26 (3.95–10.03)	9.06 (5.68–13.37)	0.002	< 0.001
PLR, median (IQR)	180 (128–261)	165 (123–223)	193 (130–276)	0.002	0.001
IVT, no./total n (%)	109 (18.6)	36 (19.0)	73 (18.4)	0.83	0.86
Prestroke mRS, no./total n (%)					
0	499 (85.3)	167 (88.4)	332 (83.8)		
1	60 (10.3)	18 (9.5)	42 (10.6)		
2	26 (4.4)	4 (2.1)	22 (5.6)		
Baseline NIHSS score, median (IQR)	27 (17–33)	18 (11–27)	30 (21–34)	< 0.001	< 0.001
Baseline pc-ASPECTS score, median (IQR)	8 (7–9)	9 (8–10)	7 (6–9)	< 0.001	< 0.001
PC-CS score, median (IQR)	5 (3–6)	5 (4–6)	4 (3–6)	0.08	< 0.001
Occlusion sites, no./total n (%)				0.002	0.002
BA distal	194 (33.2)	83 (43.9)	111 (28.0)		
BA middle	185 (31.6)	50 (26.5)	135 (34.1)		
BA proximal	97 (16.6)	29 (15.3)	68 (17.2)		
VA-V4	109 (18.6)	27 (14.3)	82 (20.7)		
Stroke etiology, no./total n (%)				0.90	0.03
Large artery atherosclerosis	381 (65.1)	109 (57.7)	272 (68.7)		
Cardioembolism	152 (26.0)	60 (31.7)	92 (23.2)		
Others	52 (8.9)	20 (10.6)	32 (8.1)		
Anesthesia, no./total n (%)	223 (38.1)	64 (33.9)	159 (40.2)	0.18	0.14
First pass, no./total n (%)	303 (56.7)	123 (69.9)	180 (50.3)	0.04	< 0.001
Workflow times, min, median (IQR)					
Onset to imaging time	209 (85–356)	200 (86–317)	210 (85–364)	0.74	0.68
Onset to puncture time	246 (130–390)	244 (132–355)	246 (129–408)	0.76	0.77
Puncture to recanalization time	105 (71–151)	85 (60–126)	115 (80–162)	0.001	< 0.001
mTICI, no./total n (%)				< 0.001	< 0.001
0-2a	107 (18.3)	11 (5.8)	96 (24.2)		
2b-3	478 (81.7)	178 (94.2)	300 (75.8)		

*Abbreviations*: *IQR* interquartile range, *NLR* neutrophil-to-lymphocyte ratio, *PLR* platelet-to-lymphocyte ratio, *NIHSS* National Institutes of Health Stroke Scale, *pc-ASPECTS* posterior- circulation Alberta Stroke Program Early CT Score, *PC-CS* posterior circulation collateral score, *BA* basilar artery, *VA-V4* vertebral artery-V4 segment, *mTICI* modified postprocedural Thrombolysis in Cerebral Infarction

^*^Adjusted estimates of effect were calculated using multiple regression taking the following variables into account: age, diabetes mellitus, puncture to recanalization time, NIHSS, pc-ASPECTS, occlusion sites, TOAST

We excluded patients with missing essential data from the analysis; therefore, we did not impute for missing data. Differences with *P* < 0.05 were considered statistically significant, and all hypothesis tests were two-sided. Statistical analyses were performed using SPSS (version 26; IBM Corp., Armonk, NY, USA), and figures were generated using STATA (version 16.0; StataCorp LLC, TX) and Excel 2019 (Microsoft Corp.).

## Results

### Patient characteristics

**Fig. 1:**
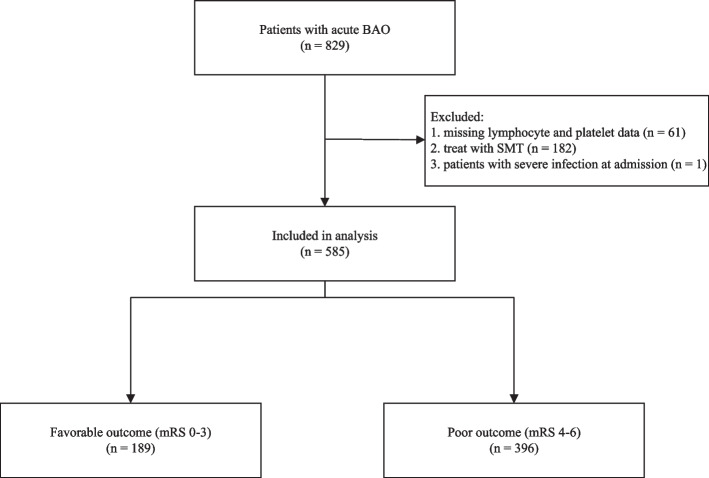
Flow diagram of the present study. BAO: basilar artery occlusion; SMT: standard medical treatment; mRS: modified Rankin Scale

Of the 829 patients enrolled in the BASILAR registry, our study recruited 585 patients with acute BAO who had undergone EVT. All patients were followed up at 90 days. Of these patients, 189 experienced favorable outcomes, while 396 had poor outcomes. Figure 1 shows the flowchart of the present study.

Table 1 reports the baseline characteristics of the patients in the present study. Patients in the favorable-outcome group had a higher systolic blood pressure on admission (median [IQR]:146 [130–161] mmHg vs. 150 [134–168] mmHg; *P* = 0.048), lower NLR value (median [IQR]: 6.26 [3.95–10.03] vs. 9.06 [5.68–13.37]; *P* < 0.001), lower PLR value (median [IQR]:165 [123–223] vs. 193 [130–276]; *P* < 0.001), lower baseline NIHSS score (median [IQR]:18 [11–27] vs. 30 [21-34]; *P*<0.001), higher baseline pc-ASPECTS (median [IQR]: 9 [8–10] vs. 7 [6-9]; *P*<0.001), higher PC-CS score (median [IQR]: 146 [130–162] vs. 151 [135–169]; *P* = 0.03), and lower puncture-to-recanalization time (median [IQR]: 85 [60–126] min vs. 115 [80–162] min; *P* < 0.001) than those in the poor-outcome group. Moreover, the proportions of patients with diabetes mellitus (29 of 189 patients [15.3%] vs. 105 of 396 patients [26.5%]; *P* = 0.003) and coronary heart disease (21 of 189 patients [11.1%] vs. 71 of 396 patients [17.9%]; *P* = 0.03) in the favorable-outcome group were lower than those in the poor-outcome group. Occlusion sites (distal basilar artery: 83 of 189 patients [43.9%] vs. 111 of 396 patients [28.0%]; middle basilar artery: 50 of 189 patients [26.5%] vs. 135 of 396 patients [34.1%]; proximal basilar artery: 29 of 189 patients [15.3%] vs. 68 of 396 patients [17.2%]; and vertebral artery–V4 segment: 27 of 189 patients [14.3%] vs. 82 of 396 patients [20.7%]; *P* = 0.002) and stroke etiology (large-artery atherosclerosis: 109 of 189 patients [57.7%] vs. 272 of 396 patients [68.7%]; cardioembolism: 60 of 189 patients [31.7%] vs. 92 of 396 patients [23.2%]; and other: 20 of 189 patients [10.6%] vs. 32 of 396 patients [8.1%]; *P* = 0.03) were significantly different between the favorable-outcome and poor-outcome groups. Other baseline characteristics were not statistically different between the two groups

### NLR values, PLR values, and 90-day outcomes

Table 2 shows the predicted values of favorable outcomes and mortality of the NLR and PLR values. The rate of favorable outcomes gradually decreased with increasing NLR and PLR values; however, mortality did not exhibit a significant difference. Based on their IQRs, the NLR and PLR values were divided into four different-level groups to investigate their independent effects on favorable outcomes and mortality. After adjusting for confounders, multivariable logistic regression reported that the NLR value (adjusted OR for Q3 vs. Q1: 0.382, 95% CI: 0.232–0.631, *P* < 0.001; adjusted OR for Q4 vs. Q1: 0.340, 95% CI: 0.204–0.567, *P* < 0.001) and PLR value (adjusted OR for Q4 vs. Q1, 0.513, 95% CI, 0.307–0.856, *P* = 0.01) were significantly associated with favorable outcomes. Furthermore, the NLR value was associated with mortality (adjusted OR for Q3 vs. Q1: 2.033, 95% CI: 1.165–3.546, *P* = 0.01; adjusted OR for Q4 vs. Q1: 2.096, 95% CI: 1.197–3.671, *P* = 0.01). The predicted probabilities of favorable outcomes and mortality with NLR and PLR values are presented in Figs. S1 and S2. ROC-curve analyisis revealed AUC and optimal cutoff values of 0.628 and 7.75 for the NLR and 0.583 and 191 for the PLR, respectively (Fig. S3).

**Table 2:** Multivariable analysis: the effect of NLR and PLR values on favorable outcomes

	Favorable outcome			Mortality		
	No. of patients	Adjusted value (95% CI)*	*P* value	No. of patients	Adjusted value (95% CI)*	*P* value
NLR value at admission	189 (22.3)	0.950 (0.920–0.981)	0.002	259 (44.3)	1.017 (0.995–1.039)	0.13
Quartile						
Q1 (< 4.96)	66 (34.9)	Reference	NA	48 (18.5)	Reference	NA
Q2 (4.96–7.92)	56 (29.6)	0.795 (0.444–1.424)	0.44	63 (24.3)	1.550 (0.888–2.703)	0.12
Q3 (7.93–12.59)	35 (18.5)	0.353 (0.189–0.658)	0.001	73 (28.2)	2.033 (1.165–3.546)	0.01
Q4 (> 12.59)	32 (16.9)	0.304 (0.160–0.576)	< 0.001	75 (29.0)	2.096 (1.197–3.671)	0.01
PLR value at admission	189 (32.3)	0.997 (0.995–0.999)	0.002	259 (44.3)	1.001 (0.9998–1.002)	0.10
Quartile						
Q1 (< 128)	54 (28.9)	Reference	NA	60 (23.6)	Reference	NA
Q2 (128–180)	58 (31.0)	1.166 (0.647–2.104)	0.61	58 (22.8)	1.090 (0.630–1.885)	0.76
Q3 (181–261)	42 (22.5)	0.700 (0.379–1.292)	0.25	67 (26.4)	1.393 (0.803–2.419)	0.24
Q4 (> 261)	33 (17.6)	0.487 (0.258–0.917)	0.03	69 (27.2)	1.363 (0.792–2.346)	0.26

*Abbreviations*: *NLR* neutrophil-to-lymphocyte ratio, *PLR* platelet-to-lymphocyte ratio, *NA* not applicable

^*^Adjusted estimates of effect were calculated using multiple regression taking the following variables into account: age, diabetes mellitus, puncture to recanalization time, NIHSS, pc-ASPECTS, occlusion sites, TOAST

### The combined effect of NLR and PLR values on favorable functional outcome

**Fig. 2:**
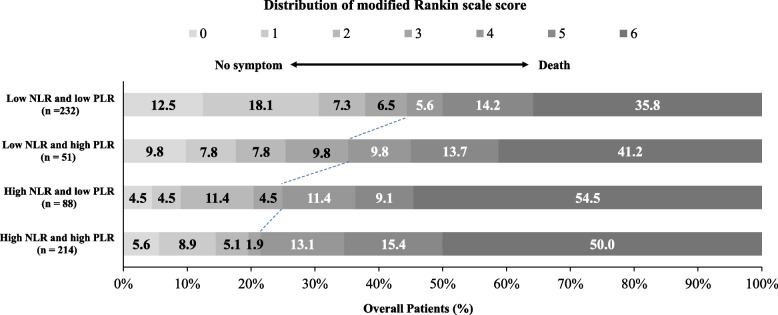
Distribution of the mRS scores at 90 days of 4 groups

Stratified analysis using the multivariable logistic regression model revealed that high NLR and PLR values (NLR value ≥ 7.75 and PLR value ≥ 191) reported the lowest rate of favorable outcomes (adjusted OR: 0.292, 95% CI: 0.173–0.494, *P* < 0.001), followed by a high NLR value alone (adjusted OR: 0.357, 95% CI: 0.183–0.695, *P* = 0.002). No obvious interaction was observed between the effects of NLR and PLR values on favorable outcomes (*P* = 0.40) (Table 3). The distribution of mRS scores across the four different NLR- and PLR-value groups are presented in Fig. 2. Furthermore, Kaplan–Meier curve analysis revealed that both high NLR and PLR values had a lower cumulative probability of survival during the 1-year follow-up (Fig. S4).

**Table 3:** Joint value of NLR and PLR on favorable outcomes in acute BAO patients after EVT

NLR ≥ 7.75	PLR ≥ 191	No. of Patients	Favorable outcome	Adjusted value (95% CI)*	*P* value
Yes	Yes	214	46 (21.5)	0.292 (0.173–0.494)	< 0.001
Yes	No	88	22 (25.0)	0.357 (0.183–0.695)	0.002
No	Yes	51	18 (35.3)	0.695 (0.323–1.495)	0.35
No	No	232	104 (44.4)	Reference	NA

*Abbreviations*: *NLR* neutrophil-to-lymphocyte ratio, *PLR* platelet-to-lymphocyte ratio, *NA* not applicable

^*^Adjusted estimates of effect were calculated using multiple regression taking the following variables into account: age, diabetes mellitus, puncture to recanalization time, NIHSS, pc-ASPECTS, occlusion sites, TOAST

### Subgroup analysis

We enrolled 166 patients without EVT from the BASILAR registry to investigate the association between NLR, PLR value and favorable outccome. In the subgroup analysis, 17 patients (10.2%) without EVT had a favorable outcome at 90 days. We found there was no significant statistical difference to indicate low NLR (unadjusted OR: 0.936, 95% CI: 0.845–1.037, *P* = 0.21) and PLR (unadjusted OR: 0.998, 95% CI: 0.993–1.003, *P* = 0.39) were both associated with favorable outcomes in patients with acute BAO without EVT (Table S1).

## Discussion

To the best of our knowledge, few studies have investigated the individual and combined prognostic values of the NLR and PLR in patients with acute BAO who have undergone EVT. Our study reports that the rate of favorable post-EVT outcomes in patients with acute BAO was 32.3%, which is comparable to that in other previous studies. In this study, we found lower NLR and PLR values to be associated with favorable post-EVT outcomes in patients with acute BAO but not in patients without EVT; furthermore, the combined value of the NLR and PLR exhibited greater efficacy in recognizing high-risk patients.

In recent years, increasing evidence has reported that inflammatory mechanisms play indispensable roles in the pathogenesis and progression of AIS. After AIS onset, numerous neutrophils are initially recruited to the ischemic-tissue area to release chemical mediators, which are associated with poor neurological improvement and worsening tissue damage, and the accumulated neutrophils potentially impair vascular remodeling during AIS recovery by producing neutrophil extracellular traps [13, 22]. Moreover, certain types of lymphocytes appear to be the major cerebroprotective immunomodulators. However, lymphocyte counts gradually decrease because of a special AIS-induced immunosuppression state [23, 24]. Additionally, platelet overactivation and accumulation potentially contribute to plaque destabilization, thus hindering AIS recovery [25]. The present study also demonstrated that the favorable-outcome group had higher neutrophil counts (*P* < 0.001) but lower lymphocyte counts (*P* = 0.01) than the poor-outcome group. No significant difference in platelet counts was noted between the two groups.

NLR and PLR values have been described as potential novel indicators of systemic inflammation intensity and reported as predictive factors of bacterial infection, thus indicating a superior predictive value to that of conventional inflammatory markers, such as neutrophils and lymphocytes [26]. Previous studies have shown higher NLR values to be related to stroke severity, poor short- and long-term outcome, unsuccessful reperfusion, and high rates of mortality [26, 27]. High PLR values have been associated with an inflamed intravascular plaque and thrombus formation [28]. Furthermore, NLR and PLR values have been widely used to predict the prognosis of cancer, cardiac disease, and sepsis [29]. The present study further confirmed that low NLR and PLR values were associated with favorable outcomes in patients with acute BAO who had undergone EVT.

PLR and NLR values have been found to be more stable than individual inflammatory markers elicited by certain physiological and pathological conditions [28]. Additionally, the combined use of PLR and NLR values has been found to depict both procoagulant and proinflammatory statuses before EVT, thus facilitating the correct selection of biomarkers. Moreover, a previous study [30] reported that the combined value of the NLR and PLR may help identify high-risk patients with AIS more effectively. A multicenter registry [28] indicated that the combined value of the NLR and PLR is potentially reliable and useful in predicting the post-EVT reperfusion grade in patients with AIS. Therefore, the combined application of NLR and PLR values is reasonable for predicting clinical post-EVT outcomes in patients with acute BAO. In the present study, our findings demonstrated that a high combined NLR and PLR value exhibited the highest risks of poor outcomes and mortality compared with low combined values. Figure 2 shows that the distribution of mRS scores at 90 days for high combined NLR and PLR values was essentially shifted toward worse outcomes. Furthermore, based on Kaplan–Meier curve analysis (Fig. S4), patients with the highest combined NLR and PLR values exhibited significantly higher rates of mortality at 1-year follow-up than those in other groups. A possible reason for these findings may be the divergent influences of NLR and PLR values. NLR values predominantly represent inflammatory damage, whereas PLR values have thrombotic and inflammatory effects. Overall, the combined application of NLR and PLR values potentially facilitates the prediction of clinical post-EVT outcomes in patients with acute BAO.

This study has certain limitations. First, the present study was retrospectively biased due to the study design. Second, since the BASILAR was a multicenter observational study, the post-EVT outcomes might have been affected by procedure-related factors, including the operator’s technique and expertise, among others, and these factors were not acquirable for analysis in the present study. Third, blood samples were exclusively collected upon admission. Hence, without continuous measurements, the present study could not confirm the relationships between these biomarkers and post-EVT outcomes. Despite these limitations, the findings of the present study provide insight into the association of NLR and PLR values with favorable functional outcomes in patients with acute BAO after EVT.

## Conclusions

In conclusion, this study’s findings suggest that low NLR and PLR values are associated with favorable outcomes in patients with acute BAO who underwent EVT. Moreover, the combined value of the NLR and PLR is useful and reliable in predicting clinical post-EVT outcomes. To prove these findings and guide the clinical application of NLR and PLR values, further studies are warranted.

## Supplementary Information

**Additional file 1: Table S1.** The association between NLR, PLR value and favorable outcome. **Figure S1.** Predicted probability of favorable outcome by neutrophil to lymphocyte ratio, platelet to lymphocyte ratio. **Figure S2.** Association of predicted value of favorable outcome between neutrophil to lymphocyte ratio and platelet to lymphocyte ratio. **Figure S3. **Receiver operating characteristic curves. **Figure S4.** The Kaplan-Meier curve.

## Data Availability

The datasets used and/or analysed during the current study available from the corresponding author on reasonable request.

## Abbreviations

NLR: Neutrophil-to-lymphocyte ratio
PLR: Platelet-to-lymphocyte ratio
BAO: Basilar artery occlusion
EVT: Endovascular treatment
OR: Odds ratio
CI: Confidence interval
