# Supplementary material for: Low neutrophil-to-lymphocyte and platelet-to-lymphocyte ratios predict favorable outcomes after endovascular treatment in acute basilar artery occlusion: subgroup analysis of the BASILAR registry
Source: BMC Neurol. 2023 Mar 20;23:113. doi: 10.1186/s12883-023-03161-2 (PMC10026508; doi:10.1186/s12883-023-03161-2)
Supplement: Supplementary file 1 — Additional file 1: Table S1. The association between NLR, PLR value and favorable outcome. Figure S1. Predicted probability of favorable outcome by neutrophil to lymphocyte ratio, platelet to lymphocyte ratio. Figure S2. Association of predicted value of favorable outcome between neutrophil to lymphocyte ratio and platelet to lymphocyte ratio. Figure S3. Receiver operating characteristic curves. Figure S4. The Kaplan-Meier curve. [file 12883_2023_3161_MOESM1_ESM.docx]

**Supplement Material**

Table S1: The association between NLR, PLR value and favorable outcome.

|  | Unadjusted Value (95% CI) | P value |
| --- | --- | --- |
| NLR | 0.936 (0.845-1.037) | 0.21 |
| PLR | 0.998 (0.993-1.003) | 0.39 |

Figure S1: Predicted probability of favorable outcome by neutrophil to lymphocyte ratio, platelet to lymphocyte ratio.**
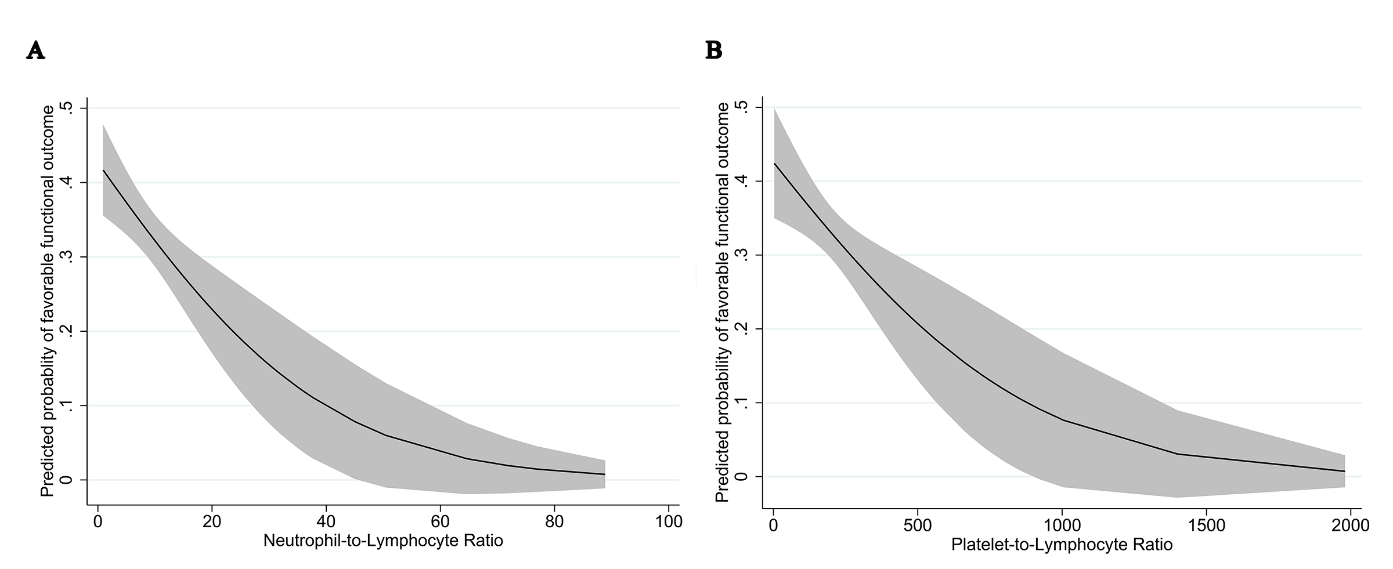
**

Figure S2: Association of predicted value of favorable outcome between neutrophil to lymphocyte ratio and platelet to lymphocyte ratio.

**
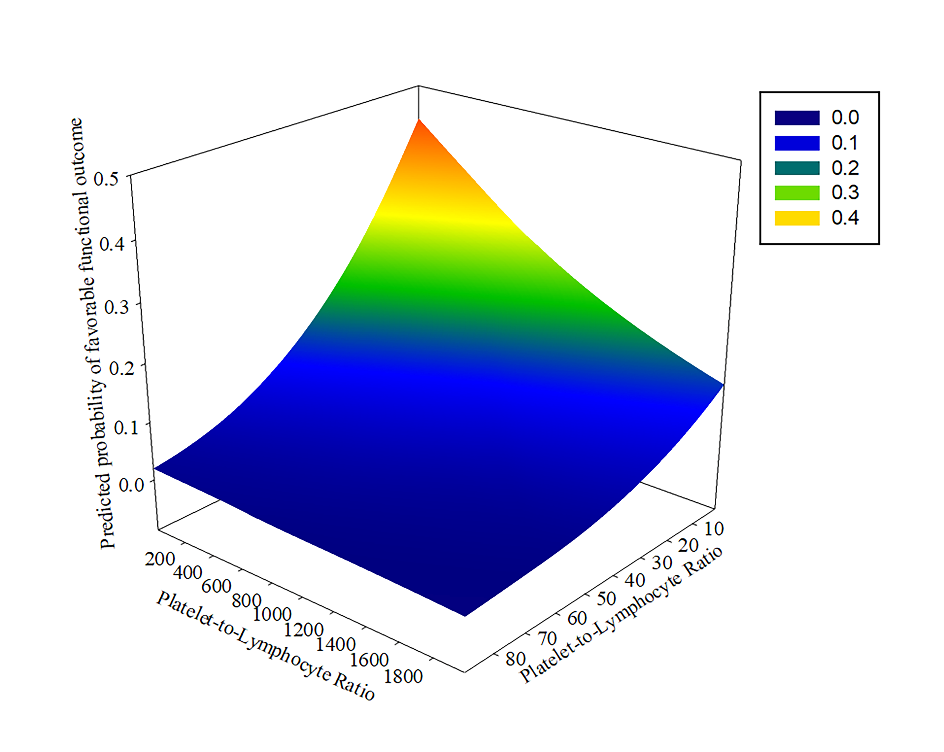
**

Figure S3: Receiver operating characteristic curves

**
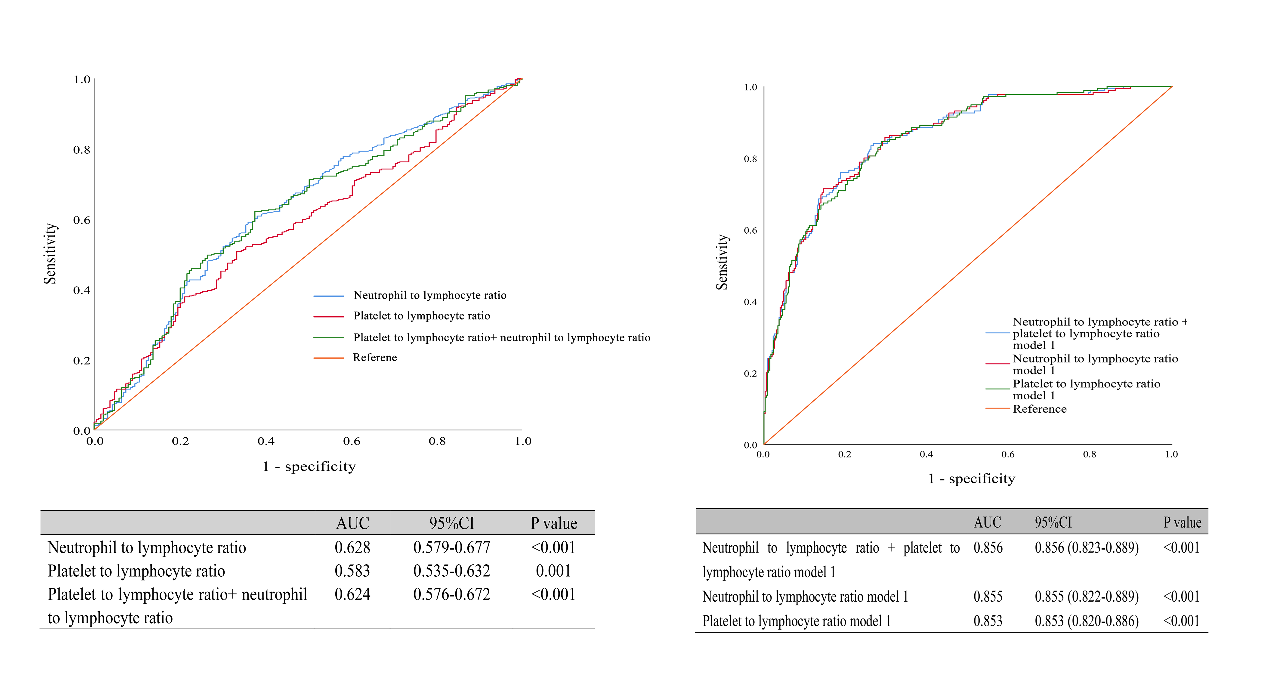
**

Figure S4：The Kaplan-Meier curve

**
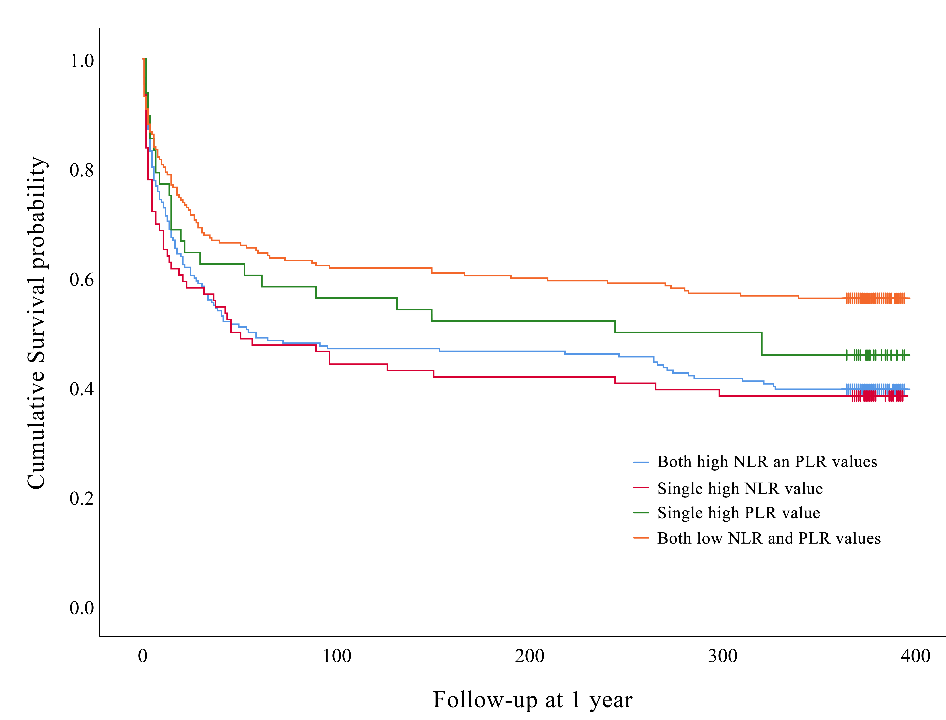
**
